# Recurrent vulvar ulcers led to the multidisciplinary management of a rare vulvar condition, Langerhans cell histiocytosis

**DOI:** 10.1016/j.jdcr.2025.11.008

**Published:** 2025-11-10

**Authors:** Shubham Kalwani, Shira Fishbach, Alexander S. Taylor, Ebony C. Parker-Featherstone, Asra Ahmed, Natalie A. Saunders, Kathryn C. Welch

**Affiliations:** a Department of Obstetrics & Gynecology, Michigan Medicine, University of Michigan, Ann Arbor, Michigan; b Department of Family Medicine, Michigan Medicine, University of Michigan, Ann Arbor, Michigan; c Department of Pathology, Michigan Medicine, University of Michigan, Ann Arbor, Michigan; d Boyce and Bynum Pathology Professional Services, Columbia, Missouri; e Division of Hematology/Oncology, Department of Internal Medicine, University of Michigan, Ann Arbor, Michigan

**Keywords:** gynecology, histiocytosis, hydroxyurea, Langerhans cells, rare diseases, vulva, vulvar diseases

## Introduction

Langerhans cell histiocytosis (LCH) is a rare disorder characterized by the expansion of myeloid precursors. It presents in a continuum of involvement from a solitary cutaneous lesion to multisystem organ dysfunction.^1^ Definitive diagnosis is typically achieved through tissue biopsy demonstrating a clonal neoplastic proliferation with characteristic immunohistochemical markers.^1^^,^^2^ LCH most commonly affects children, with the annual incidence reported to be 2.6 to 8.9 cases per 1 million children compared with an estimated incidence of 0.07 cases per million in adults. The skeleton is the most affected organ system with a range of skin involvement also being relatively common, including the mucosa of the oral cavity and vulvar tissue.^1^

LCH affecting the vulva is exceedingly rare with only a handful of case reports in the published literature. Vulvar involvement has typically been described as pruritic ulcerations, erythematous plaques, indurated nodules, or papular lesions.^3^ There is no established standard of care for vulvar LCH, with treatment options including surgical excision, radiotherapy, chemotherapy, and thalidomide.^1^^,^^2^ The vulva is rarely the primary site and additional workup commonly reveals disseminated disease. The underlying disease process can be aggressive with many patients experiencing local recurrence or metastasis despite treatment.

## Case report

**Fig 1:**
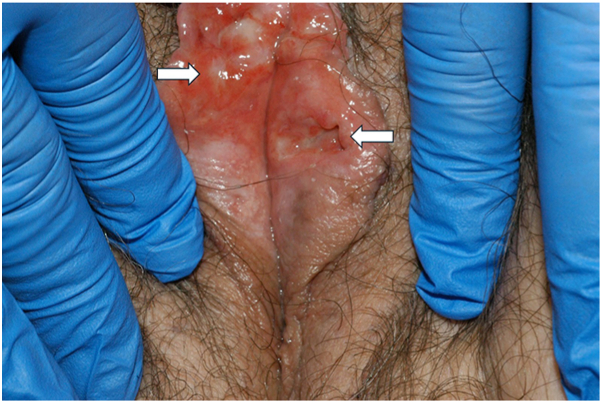
Erosion with surrounding erythema of the right labium minus (*right arrow*) and full thickness perforation of the left labium minus (*left arrow*).

**Fig 2:**
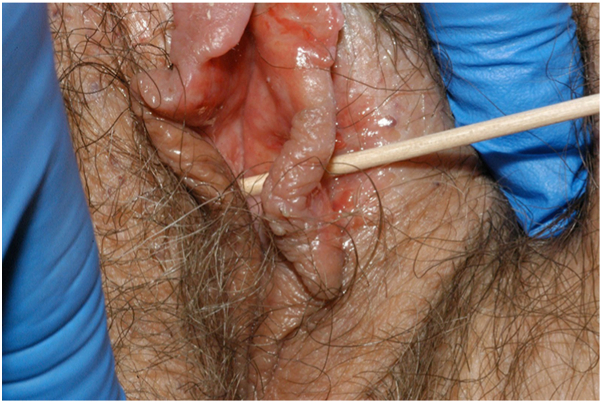
Ulceration extending into a full thickness perforation of the left labium minus.

We describe a case in which a patient presented with recurrent vulvar erosions, ulcerations, and full-thickness perforations of the left labium minus (Figs 1 and 2). Evaluation ultimately resulted in a diagnosis of LCH. The vulvar lesions were a key clue to underlying disseminated disease, with metastatic workup revealing the involvement of bone, breast, GI tract, and right external auditory canal. We detail the initial diagnostic challenge, multidisciplinary collaboration, as well as this patient’s ongoing clinical course to shed light on this rare disease.

Our patient, a 63 year-old postmenopausal female, presented for evaluation of recurrent vulvar erosions and ulcerations to our clinic in the fall of 2021. Her symptoms started in 2017 initially with vulvar irritation and urinary retention. Her past medical history was significant for diabetes insipidus and hip arthroplasty. A vulvar biopsy in 2018 was non-specific. Prior treatment consisted of topical corticosteroids, which provided only partial symptom relief. Initial diagnoses considered included infection, neoplasm, plasma cell vulvitis, and Behçet disease.^4^ However, the International Criteria for Behçet were not met. An extensive infectious disease evaluation was negative. Due to worsening symptoms, a repeat biopsy revealed vulvar ulceration with a dense plasma cell infiltrate. Immunohistochemical testing was negative for HSV 1, HSV 2, and spirochetes. Direct immunofluorescence testing was negative for immune deposits.

**Fig 3:**
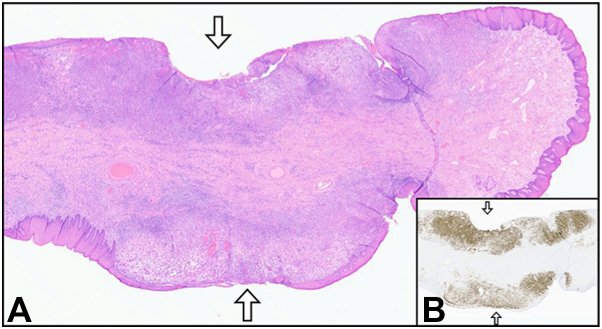
**A,** Vulvar skin (hematoxylin and eosin stain) with multifocal ulceration (*arrows*) and underlying edema, inflammation, and neoplastic cellular infiltrate. **B,** Same tissue section (*arrows* at same sites of ulceration) with dense and diffuse band-like infiltrate of Langerhan cells highlighted by Langerin immunohistochemical stain (brown). 10× magnification **(A** and **B)**.

**Fig 4:**
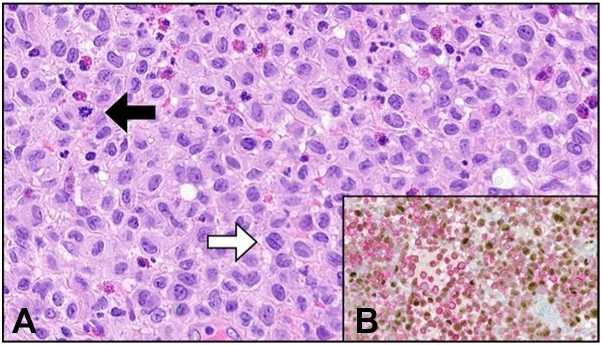
**A,** Confluent sheets of neoplastic mononuclear cells (hematoxylin and eosin stain) with eosinophilic cytoplasm and prominent nuclear grooves/folds (*white arrow*), fine chromatin, indistinct nucleoli, and mitotic activity (*black arrow*). **B,** Immunohistochemical dual stain for cyclin-D1 (brown, nuclear staining) and CD1a (pink/red, membranous/cytoplasmic staining) demonstrates co-expression of both proteins in the lesional cells, consistent with a neoplastic Langerhan cell population. 400× magnification **(A** and **B)**.

Since the gross appearance of the vulva was not consistent with plasma cell vulvitis, her case was re-reviewed for additional clinicopathological correlation, and a component of Langerhans cells was noted among the plasma cells. Additional immunohistochemical stains were performed and showed co-expression of CD1a and Cyclin D1 in the Langerhans cell population, consistent with a diagnosis of Langerhans cell histiocytosis (Figs 3 and 4).^5^

Further workup demonstrated biopsy-proven involvement of bone, stomach, colon, and esophagus. Additionally, she had presumed involvement of the right external auditory canal, breast, and oral cavity based on symptoms as well as a PET scan. After years of suffering with unexplained symptoms, she was treated with 10 cycles of cytarabine. Despite the regression of all other sites, she had refractory vulvar disease. At this time, full-thickness ulcers had progressed, resulting in significant pain. Surgical excision of the affected tissue was offered and performed in March 2023, with excellent healing. However, relief was short-lived and her vulvar lesions returned within months.

The patient’s oncology team collaborated nationally to address her ongoing symptoms. After discussion, it was recommended to first try hydroxyurea for her persistent vulvar disease. If this did not work to control her vulvar disease, the next recommendation was to initiate thalidomide or revlimid. She was subsequently started on hydroxyurea 500 mg by mouth daily. There had been some variation in dosing, with brief periods on 1000 mg daily, but overall she has been maintained on 500 mg daily. About 6 months after treatment initiation, she had significant improvement of her symptoms and reduction of lesions on exam. At 12 months, there was a decrease in disease burden of the vulva on PET. On a repeat PET scan 3 months later, there was complete resolution of vulvar disease. Concurrent examination at this time, 15 months after treatment initiation, revealed complete resolution of her vulvar disease on exam. She continues to receive treatment with hydroxyurea and remains under surveillance.

## Discussion

Vulvar erosions and ulcerations are common presenting complaints in a clinical setting with a broad differential diagnosis. Although LCH is quite rare, if it is not considered in the differential of vulvar lesions, a delay in diagnosis and treatment is likely. For this patient, this led to years of difficult symptomatology.

This highlights the importance of clinicians in multiple specialties, including gynecology and primary care, to recognize this condition, especially in refractory cases. This case also emphasizes the potential need for multiple biopsies in recurrent cases of ulceration. Genital ulcers biopsies are often non-specific which increases diagnostic complexity. A retrospective study by Chan et al reviewed 98 cases of genital ulcer biopsies and found almost 20% of patients required repeat biopsies.^6^ A similar presentation achieved diagnostic clarity with additional biopsy.^7^ Specifically for biopsy of a vulvar ulcer, it can be helpful to biopsy the ulcer edge.^8^

Regarding treatment, a multidisciplinary approach is needed. Many of these cases will have recurrence of disease. In the majority of case reports reviewed for vulvar LCH, thalidomide was often noted as a successful treatment option. In this case, we offered hydroxyurea as another option for treatment for her refractory disease. Clinical evidence supports the use of hydroxyurea in multisystem disease and for anogenital lesions. Hydroxyurea also has a more favorable side effect profile compared to thalidomide.^1^^,^^2^ Further studies are needed regarding length of treatment and long-term outcomes.

## Conflicts of interest

None disclosed.
